# Dystrophic Adipocytes Mimicking Metastatic Signet Ring Cell Adenocarcinoma: A Diagnostic Pitfall in a Cachectic Patient

**DOI:** 10.1155/2018/9027870

**Published:** 2018-05-07

**Authors:** Xin Zhang, Jennifer J. Findeis-Hosey

**Affiliations:** Department of Pathology and Laboratory Medicine, University of Rochester Medical Center, P.O. Box 626, 601 Elmwood Ave, Rochester, NY 14642, USA

## Abstract

Cachexia is a debilitating condition and complex syndrome commonly associated with a variety of chronic diseases. It is caused by metabolic dysregulation and characterized by profound loss of adipose tissue and skeletal muscles. While pathological changes of cachectic conditions on adipose tissue have been studied and documented in tumor-bearing animal models, similar morphological changes in human surgical specimens are rare. Here we report a case of a cachectic patient with pancreatic adenocarcinoma whose adipocytes underwent dramatic lipodystrophy mimicking signet ring cell adenocarcinoma. The patient had presented with a large bowel obstruction, a mass extending between the pancreas and colon, and radiographic concern for carcinomatosis. A moderately differentiated adenocarcinoma was identified invading externally into the colon, with extensive signet ring-like cells throughout the specimen, including those adjacent to the colon and lymph nodes and around nerves. These signet ring-like cells were round with variably clear to eosinophilic cytoplasm and a peripherally displaced round to oval nucleus. Immunohistochemical staining demonstrated that these signet ring-like cells were negative for AE1/AE3, CD138, or Kreyberg staining, while they were positive for S-100 staining, confirming these as dystrophic adipocytes. Here we examine dystrophic adipocytes in a cachetic patient, examining the differential diagnosis and potential ancillary studies.

## 1. Introduction

Profound loss of adipose tissue is the hallmark of cachexia. It is estimated that approximately 20% of deaths in cancer patients are due to cachexia [1]. Prolonged starvation, malnutrition, and changes in metabolic processes cause tissues and organs to undergo atrophy. Although the exact molecular and cellular mechanisms underlying the histological changes of adipose tissue atrophy are not fully understood, studies have suggested that it results from the combination of increased metabolic processes and impaired anabolic function [2, 3]. Despite the atrophic changes of the tissue, the morphology of dystrophic organs and cells is usually still recognizable histologically. The remodeling of adipose tissue causes adipocytes to decrease in size, with increased size variation and a more rounded shape. The atrophic adipose tissue generally maintains a lobulated structure without infiltration and the dystrophic cells are separated by myxoid or mucoid stroma with fibrosis and a delicate vascular network. Here we examine a case of a cachetic patient with widespread atrophy and signet ring-like cellular morphology of adipose tissues, raising the differential diagnosis of metastatic signet ring cell carcinoma.

## 2. Case Presentation

The patient was a 63-year-old male former smoker with a history of insulin dependent diabetes mellitus and hypothyroidism. He reported having a “sensitive stomach” for three years with cyclical episodes of nausea, vomiting, diarrhea, constipation, and poor oral intake over the course of three months, with progressive weakness and a 40 lb weight loss. Initially his symptoms were treated conservatively based on imaging demonstrating a diffusely distended GI tract concerning for enterocolitis or ileus. Conservative management was unsuccessful, with the patient presenting to the emergency department due to the sudden onset of intractable severe vomiting that lasted for hours, coupled with severe abdominal distention. The patient had never previously had a colonoscopy or esophagogastroduodenoscopy.

Physical examination revealed a cachectic man (BMI 16.69 kg/m^2^, Glasgow prognostic score 1 with intermediate prognosis) with a mildly distended and thinned abdominal wall. The abdomen was nontender with normal bowel sounds and without rebound, guarding, or evidence of peritonitis. Colonoscopy demonstrated a dilated colon with friable mucosa but no evidence of ischemia. The distal transverse colon demonstrated luminal narrowing with the suggestion of extrinsic compression. A CT abdomen and pelvis with contrast showed a large bowel obstruction with a transition at the splenic flexure with focal wall thickening. Radiographically this was considered to likely be secondary to a pancreatic tail cancer with local malignant extension into the colon. Additionally, possible infiltration of the omentum in the right hemiabdomen was concerning for omental carcinomatosis. Diffuse heterogeneous bone densities raised concern for metastatic disease versus patchy osteopenia.

The patient underwent a subtotal colectomy with ileostomy. Intraoperatively it was noted that the patient was cachectic with minimal body fat. The transverse colon was congested, distended, cyanotic, and densely adherent to the retroperitoneum in the area of the pancreatic body and tail. Firm, white tissue was identified outside the pancreas with likely invasion of the middle colic vessels. Palliative subtotal colectomy and end ileostomy were performed for symptomatic relief.

On macroscopic examination, a 5.5 cm constricted area was identified in the transverse colon with associated serosal puckering. Sectioning of the puckered area revealed a firm yellow-tan cut surface and numerous submucosal cystic spaces ranging from 0.1 to 0.5 cm in greatest dimension. Sparse focally adherent pericolic fat with scattered fibrinous adhesions and palpable lymph node candidates were identified.

**Figure 1:**
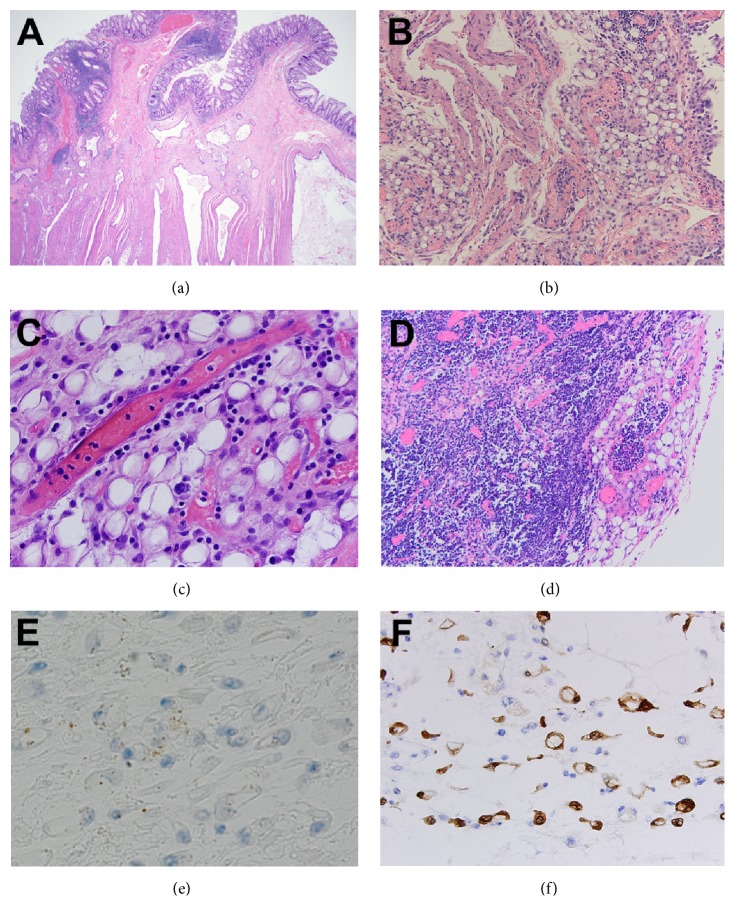
Dystrophic adipocytes mimicking metastatic signet ring cell adenocarcinoma in a cachectic patient. (a) Moderately differentiated adenocarcinoma invading externally into the colon at the splenic flexure with associated colonic stricture, ulceration, and mural fibrosis (hematoxylin-eosin stain, ×200). (b, c) The pericolonic tissue, mesentery, subserosa, and lymph nodes demonstrated signet ring-like cells that showed variation in size with a thickened cell membrane (hematoxylin-eosin stain, (b) ×100, (c) ×400). (d) Pericolonic lymph node demonstrated signet ring-like cells mimicking the appearance of metastatic involvement (hematoxylin-eosin stain, ×100). (e, f) Immunohistochemical staining of the signet ring-like cells demonstrates these cells to be negative for AE1/AE3 staining ((e) ×400), while being positive for S-100 staining ((f) ×400).

Histologic examination demonstrated a moderately differentiated adenocarcinoma invading externally into the colon at the splenic flexure with associated colonic stricture, ulceration, and mural fibrosis (Figure 1(a)). The pericolonic tissue, mesentery, subserosa, and lymph nodes demonstrated signet ring-like cells without typical adipocytes (Figures 1(b) and 1(c)). The signet ring-like cells demonstrated a round to oval shaped nucleus which was pushed to the side of the cell by a cleared out cytoplasmic vacuole, resembling a signet ring. The cytoplasm was variably clear to eosinophilic. These signet ring-like cells were smaller in comparison to typical mature adipocytes and showed variation in size with a thickened cell membrane. No cellular atypia or mitotic figures were observed.

The signet ring-like cells were surrounded and separated by a mucoid to myxoid fibrovascular stroma. In the omentum, the signet ring-like cells were organized in lobulated aggregates separated by fibrous tissue. In the subserosal region, focal areas of signet ring-like cells were identified surrounding nerves, raising the concern for perineural invasion. Additionally, one pericolonic lymph node demonstrated signet ring-like cells mimicking the appearance of metastatic involvement (Figure 1(d)).

Immunohistochemical staining of the signet ring cells demonstrated these cells to be negative for AE1/AE3, CD138, or Kreyberg staining while being positive for S-100 staining (Figures 1(e) and 1(f)); the immunoprofile was most consistent with atrophic adipocytes rather than signet ring cell carcinoma.

## 3. Discussion

The term signet ring cell was originally used in the pathology literature to describe the morphology of adenocarcinomas where the nucleus has been peripherally displaced by an accumulation of cytoplasmic mucin, thus resembling the shape of a signet ring. However, a variety of benign conditions and malignant neoplasms beyond signet ring cell adenocarcinoma have now been reported with signet ring-like cells, thus leading to a differential diagnosis with potential diagnosis pitfalls. Nonneoplastic mimickers of signet ring cell adenocarcinoma have been described in gastric xanthomas [4], transurethral prostatectomy specimen [5], and pseudomembranous colitis [6]. While the majority of the reported cases of signet ring-like cells in the gastrointestinal tract involve epithelial cells that are adjacent to ischemic or injured areas, signet ring-like cellular changes involving nonepithelial cells are rare. To the best of our knowledge, signet ring-like changes have only been reported in muciphages [7], schwannomas [8], two cases involving subserosal adipocytes (one being associated with chronic ischemic enteritis [9] and the other in the setting of clostridium difficile colitis [6]), and two cases of starvation-induced fat atrophy involving the omentum [10]. Here we report a case of a signet ring cell-like change involving adipocytes in a cachetic patient diagnosed with locally aggressive pancreatic adenocarcinoma with the concern for carcinomatosis. Signet ring shaped adipocytes were identified throughout the specimen, including those adjacent to the bowel, within the omentum and lymph nodes, and around nerves. The clinicopathologic features of this case raised the concern for diffuse signet ring cell adenocarcinoma, especially given the lack of available examination of the primary pancreatic mass due to the palliative nature of the colectomy.

Similar to previous literature regarding atrophic adipocyte mimickers [10], the fat cells in this case are smaller than normal mature adipocytes and show slight variations in size and shape. They are arranged in circumscribed sheets and lobules with fibrous tissues and delicate vasculature structures. However, our case shows an infiltrating pattern in the submucosal area where the signet ring-like cells are interspersed with chronic inflammatory cells and blood vessels and adjacent to nerves, raising the concern for perineural invasion. Similar to Khan and Ligatos' report of atrophic adipocytes in the setting of clostridium difficile colitis [6], our case also shows necrotizing changes and areas of ischemia, characterized by tan-maroon colonic serosa with tan white shaggy fibrous exudate and areas of transversely oriented linear ulcerations throughout the colonic mucosa.

Diagnosing benign signet ring-like change by morphology alone can be difficult and may require ancillary studies to identify the histological origin of the signet ring-like cells. This distinction is critical for excluding malignant conditions such as primary or metastatic signet ring cell carcinoma and other malignant conditions mimicking signet ring cell adenocarcinoma. Pena et al. reported a case of mucophagocytizing histiocytes in a low grade appendiceal mucinous neoplasm mimicking signet ring cell mucosecreting adenocarcinoma [11]. Lortscher et al. reported two cases of primary cutaneous squamous cell carcinoma and basal cell carcinoma showing signet ring cell-like morphology [12]. For cases involving epithelial cells, besides identifying the cell origin, P53 and Ki67 can be helpful in distinguishing benign and malignant conditions [13].

While the exact mechanism causing cells to undergo morphological changes that mimic signet ring cells is still largely unknown, there are hypotheses that this may be caused by cytoplasmic accumulation of various substances, cell degeneration with cytoplasmic vacuole formation, cytoplasmic membrane invagination, endoplasmic reticulum dilatations, or processing artifact [12]. Bing et al. studied the potential mechanism in adipocytes using cancer-bearing cachexia mouse models and found that cachectic mouse adipose tissue demonstrated similar morphological changes as to what was observed in atrophic human adipocytes [3, 14]. They found that inhibited adipocyte development and lipid deposition contributed to the morphological change and their hypothesis is supported by the observed major reductions in mRNA levels of adipogenic transcription factors, their transcription products, and the enzymes and molecules involved in adipose tissue formation. They identified that the mRNA levels of peroxisome proliferators-activated receptor gamma coactivator-1 alpha (PCG-1*α*) and uncoupling protein-2 (UCP-2) were increased, suggesting that there is an impairment in lipid formation and storing capacity in cachetic mice. They also studied the role of lipolysis in the underlying fat loss and adipocyte morphological change. Their studies demonstrated that TNF*α* is linked with increased lipolysis, and zinc-*α*_2_-glycoprotein (ZAG), which has been identified as a novel adipokine, is upregulated in cancer cachexia which promotes lipid breakdown and can potentially serve as a novel target for preventing adipose atrophy in malignant conditions. Additionally, M. Seelaender's group has studied the subcutaneous adipose tissue architectural changes in gastrointestinal cancer patients and found that fibrosis and inflammatory cell infiltration with altered TGF-ß signaling are associated with cachetic conditions [15, 16] and has suggested that increased adiponectin, liver-derived CRP, and plasma IL-6 levels have significant correlation with the presence of cachexia and cancer [17].

In conclusion, cachexia is a debilitating condition that is commonly associated with chronic diseases, especially in cancer patients. Here we demonstrate a signet ring cell-like change observed in adipocytes in a cachectic patient. This morphologic change was so dramatic that it mimicked the histologic appearance of signet ring cell adenocarcinoma, including intimate association with nerves and lymphoid tissue. Here the careful correlation between clinicopathologic features and immunohistochemical staining was necessary for the correct identification and interpretation.

## References

[1] Tisdale M. J. (2002). Cachexia in cancer patients.

[2] Agustsson T., Rydén M., Hoffstedt J. (2007). Mechanism of increased lipolysis in cancer cachexia.

[3] Bing C., Trayhurn P. (2009). New insights into adipose tissue atrophy in cancer cachexia.

[4] Drude R. B., Balart L. A., Herrington J. P., Beckman E. N., Burns T. W. (1982). Gastric xanthoma: Histologic similarity to signet ring cell carcinoma.

[5] Alguacil-Garcia A. (1986). Artifactual changes mimicking signet ring cell carcinoma in transurethral prostatectomy specimens.

[6] Khan O., Ligato S. (2017). Identification of Signet Ring Cell Change in Colonic Subserosa in the Setting of Clostridium difficile Colitis: Report of a Nonneoplastic Mimicker of Signet Ring Cell Carcinoma.

[7] De Petris G., Lev R., Siew S. (1998). Peritumoral and nodal muciphages.

[8] Trivedi A., Ligato S. (2013). Microcystic/Reticular schwannoma of the proximal sigmoid colon: case report with review of literature.

[9] Houghton O., Herron B. (2006). Benign signet ring cells in the subserosa of the small intestine: a pseudoneoplastic phenomenon.

[10] Vajpeyi R., Chetty R. (2013). Starvation-induced fat atrophy in the omentum: A diagnostic pitfall.

[11] Pena G. P., Berenstein C. K., Ribeiro C. A. (2014). Mucophagocytizing Histiocytes in a Low-Grade Appendiceal Mucinous Neoplasm Mimicking Signet-Ring Mucosecreting Adenocarcinoma Cells.

[12] Lortscher D. N., Satter E. K., Romero L. S. (2012). Signet ring-like cells: No longer a “signature” of glandular differentiation.

[13] Wang K., Weinrach D., Lal A. (2003). Signet-Ring Cell Change Versus Signet-Ring Cell Carcinoma: A Comparative Analysis.

[14] Bing C., Russell S., Becket E. (2006). Adipose atrophy in cancer cachexia: Morphologic and molecular analysis of adipose tissue in tumour-bearing mice.

[15] Batista M. L., Henriques F. S., Neves R. X. (2016). Cachexia-associated adipose tissue morphological rearrangement in gastrointestinal cancer patients.

[16] Alves M. J., Figuerêdo R. G., Azevedo F. F. (2017). Adipose tissue fibrosis in human cancer cachexia: The role of TGF*β* pathway.

[17] Batista M. L., Olivan M., Alcantara P. S. M. (2013). Adipose tissue-derived factors as potential biomarkers in cachectic cancer patients.

